# Estimating sectoral COVID-19 economic losses in the Philippines using nighttime light and electricity consumption data

**DOI:** 10.3389/fpubh.2024.1308301

**Published:** 2024-02-29

**Authors:** Ma. Flordeliza P. Del Castillo, Toshio Fujimi, Hirokazu Tatano

**Affiliations:** ^1^Tatano Lab, Department of Social Informatics, Graduate School of Informatics, Kyoto University, Kyoto, Japan; ^2^Disaster Prevention Research Institute, Kyoto University, Kyoto, Japan

**Keywords:** commercial loss, industrial loss, GDP, economic impact assessment, multiple regression, Suomi-NPP VIIRS DNB, energy consumption

## Abstract

**Introduction:**

Economic loss estimation is critical for policymakers to craft policies that balance economic and health concerns during pandemic emergencies. However, this task is time-consuming and resource-intensive, posing challenges during emergencies.

**Method:**

To address this, we proposed using electricity consumption (EC) and nighttime lights (NTL) datasets to estimate the total, commercial, and industrial economic losses from COVID-19 lockdowns in the Philippines. Regression models were employed to establish the relationship of GDP with EC and NTL. Then, models using basic statistics and weather data were developed to estimate the counterfactual EC and NTL, from which counterfactual GDP was derived. The difference between the actual and the counterfactual GDP from 2020 to 2021 yielded economic loss.

**Results:**

This paper highlights three findings. First, the regression model results established that models based on EC (adj-*R*^2^ ≥ 0.978) were better at explaining GDP than models using NTL (adj-*R*^2^ ≥ 0.663); however, combining both EC and NTL improved the prediction (adj-*R*^2^ ≥ 0.979). Second, counterfactual EC and NTL could be estimated using models based on statistics and weather data explaining more than 81% of the pre-pandemic values. Last, the estimated total loss amounted to 2.9 trillion PhP in 2020 and 3.2 trillion PhP in 2021. More than two-thirds of the losses were in the commercial sector as it responded to both policies and the COVID-19 case surge. In contrast, the industrial sector was affected primarily by the lockdown implementation.

**Discussion:**

This method allowed monitoring of economic losses resulting from long-term and large-scale hazards such as the COVID-19 pandemic. These findings can serve as empirical evidence for advocating targeted strategies that balance public health and the economy during pandemic scenarios.

## 1 Introduction

The Coronavirus Disease 2019 (COVID-19) pandemic significantly impacted global economic activities (1–5). Global economic losses surpassed USD 8 trillion from 2020 to 2021 (2, 6). Among the COVID-19-affected countries, the Philippines experienced the 13th-highest economic loss (7). This country adopted a militaristic approach to the health hazard, implementing one of the strictest and longest COVID-19 lockdowns worldwide (8). In April 2020, around 60% of the population faced Enhanced Community Quarantine (ECQ) measures that restricted gatherings and closed public transportation, schools, and non-essential businesses as a preventive measure. Consequently, the Philippines experienced an estimated 10% GDP decline in 2020 (9).

In the context of the Philippines, several studies have assessed economic losses due to COVID-19 through various methods, such as ratio analysis (10), linear probability model (11), input–output analysis (12, 13), and compartmentalized dynamic transmission (14). These studies successfully estimated a rough snapshot of the immediate economic losses due to COVID-19. However, few have examined the long-term distribution of losses across time and sectors. This could be due to the lack of high temporal resolution economic data. For example, GDP data is available only at the national and regional levels on a quarterly and annual basis, respectively.

To bridge this research gap, this paper aims to estimate the long-term distribution of the economic losses due to the pandemic across time and sectors in the Philippines using electricity consumption (EC) and nighttime light (NTL). We focus on these because strong relationships between GDP and EC (15, 16) or NTL (17–20) have been demonstrated, indicating that EC and NTL can be good proxies for economic activities. Furthermore, recent studies (21–23) used NTL to predict GDP and assess disaster impacts in the Philippines. To the best of our knowledge, no study has applied EC or NTL to estimate economic losses due to COVID-19 in the Philippines.

Employing this approach, we aim to address the following research questions. First, how much was the total, commercial, and industrial economic loss due to COVID-19 in the Philippines? And second, how were these losses distributed across time?

The remainder of this paper is organized as follows. Section 2 elaborates on the data and methods. This section details the GDP, EC, NTL, statistical, and weather datasets description and preprocessing. It also discusses the economic loss estimation, specifically the regression models employed in the paper. Section 3 presents the modeling results and loss estimates. Section 4 discusses the findings, implications, and limitations, and provides recommendations for further research.

## 2 Materials and methods

### 2.1 Data resources

**Figure 1:**
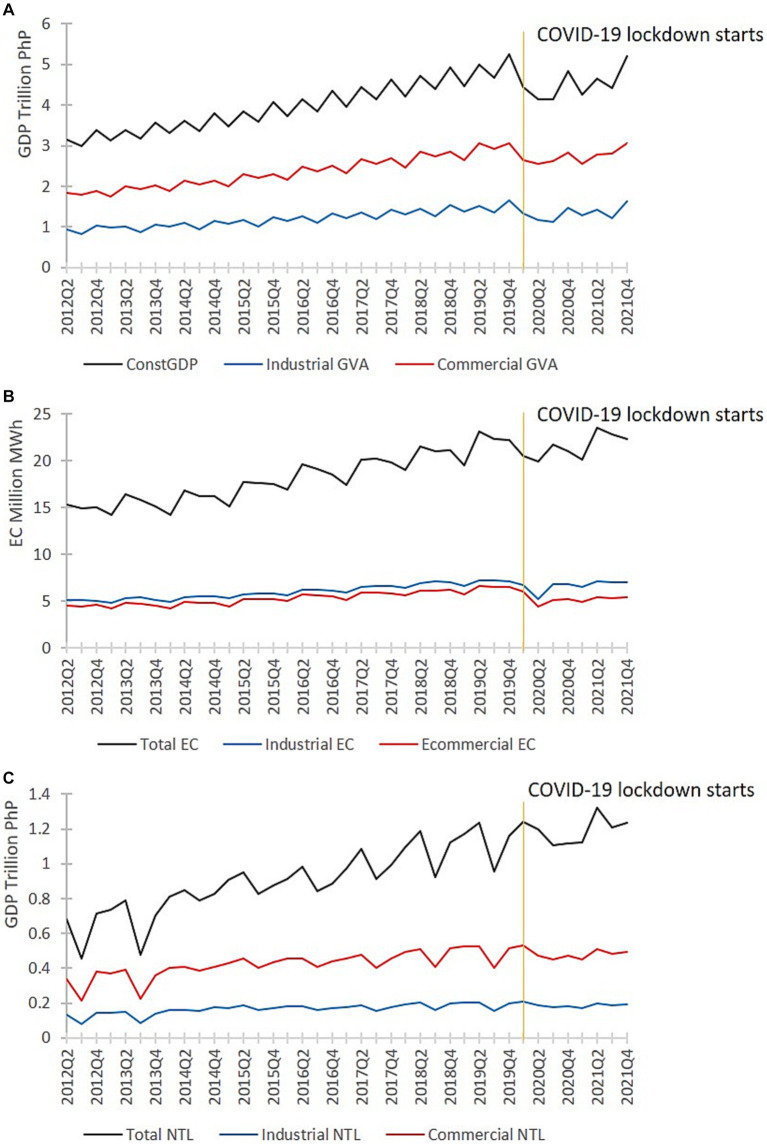
Quarterly national, commercial, and indsutrial **(A)** GDP, **(B)** EC, and **(C)** NTL data from 2012 to 2021.

Using GDP, EC, and NTL data (Figure 1), we assessed the economic losses due to COVID-19 in the Philippines. The analysis also required statistical and weather datasets. We divided the datasets into pre-pandemic (2012Q2–2019Q4) and pandemic period (2020Q1–2021Q4). We used the pre-pandemic datasets to establish the relationship between GDP, EC, and NTL and to develop EC and NTL models that estimate the pre-pandemic values based on statistical and weather datasets. Then, we used the statistical and weather data from 2020 onwards to estimate the counterfactual EC, NTL, and GDP.

#### 2.1.1 GDP

The Philippines’s GDP data covers both national quarterly and regional annual scales. In this study, we utilized the national quarterly Constant GDP data based on 2018 prices sourced from the Philippine Statistics Authority (PSA). Additionally, we collected quarterly commercial and industrial gross value-added (GVA) data to assess sectoral impacts. The commercial or services sector, encompassing businesses involved in transporting and distributing goods and services as per PSA’s classification, contributes roughly 60% to the country’s economy. Meanwhile, industries encompassing resource extraction, manufacturing, construction, energy, water, and waste management constitute approximately 30% of the country’s GDP. National GDP follows a positive trend, peaking during the fourth quarter of each year (Figure 1A). Commercial and Industrial GVAs also follow this increasing trend, with the highest GVAs during the second and fourth quarters.

#### 2.1.2 Electric consumption

The national monthly EC ( $EC_{m})$ data were sourced from the Philippine Department of Energy and downloaded through the Freedom of Information website. These data are categorized into commercial, industrial, residential, and other sectors (Supplementary Figure S1). We used the total EC data, including all the above-mentioned categories, for our analysis. We also used the commercial and industrial EC data. Commercial and industrial EC typically covered the energy used for lighting, operating electrical machines, and regulating temperature within a space. These EC data exhibit an ascending trend, with annual peaks in the second quarter, corresponding to the year’s hottest months (Figure 1B). The lowest EC occurs during the first quarter, the coldest period of the year. The monthly EC ( $EC_{m})$ was aggregated into the quarterly dataset ( $EC_{q})$ (Equation 1).


(1) $$EC_{q}=\overset{3}{\underset{m=1}{\sum }}EC_{m}\;$$

#### 2.1.3 Nighttime lights

We downloaded monthly and annual NTL composites from the Earth Observation Group of the Payne Institute for Public Policy, Colorado School of Mines.^1^ These products were preprocessed datasets from the Suomi National Polar-orbiting Partnership (NPP), Visible and Infrared Imaging Suite’s (VIIRS) Day and Night Band (DNB). The datasets underwent filtering steps to remove extraneous light sources. Filtered datasets were composited into monthly and annual radiance averages (24).

Some pixel values or Digital Numbers (DN) were negative. In theory, NTL’s DN values should start from zero, signifying the absence of light. However, negative values could be due to background noise (25). These negative values had to be removed as these could falsely reduce the sum of lights (SOL). We eliminated the negative values by replacing the negative monthly radiance values with the DN from the previous year ( $DN_{t-12})$ , as in Equation 2.


(2) $$pDN_{t}=\{\begin{array}{c} <0,DN_{t-12}\\ \geq 0,\;DN_{t} \end{array}\;\;$$

We eliminated extraneous light because the main aim was to measure anthropogenic light related to economic activities. We used the stable light mask (lit_mask) to mask the background noise from the monthly radiance band. The stable light mask is a binary data where areas with stable light have a DN value of 1 and areas with unstable light have a DN value of 0. We removed unstable light by multiplying the stable light mask with the positive DN values (*pDN*), leaving only areas with stable light (*sDN*) (Equation 3).


(3) $$sDN_{t}=pDN_{t}*lit\_mask_{t}\;\;\;$$

As the study site is a tropical country where cloud cover can often contaminate nighttime light images, the effect of the clouds had to be minimized to avoid errors resulting from undetected nighttime light emission due to clouds. Hence, we identified cloud-contaminated pixels using the cloud-free coverage band (cf_cvg band), obtaining the number of cloud-free data collected within the month. Then, we replaced the cloud-covered pixels (pixels with 0 cf_cvg values) with the pixel value of the previous year ( $sDN_{t-12})$ (Equation 4). Although complete cloud removal is impossible, especially during rainy months, this step minimizes the undetected light due to cloud cover.


(4) $$cf\_cvg=\{\begin{array}{c} \;0,\;sDN_{t-12}\\ >0,sDN\; \end{array}\;\;\;$$

We attributed the NTL source to commercial and industrial sectors for analysis. Classifying commercial and industrial NTL sources required a national land use map. However, land use maps with commercial and industrial classifications were only available for cities or provinces. An alternative, OpenStreetMap (OSM), offered a countrywide land use map with industrial and commercial classes. This dataset is a product of voluntary contributions and has been used for various applications (26). Although this data does not comprehensively cover all buildings, recent research indicates improved completeness, particularly in major cities. In the Philippines, OSM data’s completeness ranges from 20% to over 80%, exceeding 80% for major cities (26). This OSM data is the best alternative as it adequately indicates the general location of commercial and industrial sources of NTL.

We visually examined the downloaded OSM shapefiles and determined that the data were complete and accurate. Since the data would specify the country’s commercial and industrial NTL emissions, we believed its quality was satisfactory. NTL data has a ground resolution of roughly 500 meters, meaning each cell could contain a mixture of land uses. It would not be possible to ascertain the ultimate source of light detected within a 500 × 500-meter area. Hence, an indicative presence of commercial or industrial establishments would be enough.

We extracted all the points and polygons classified as commercial or industrial, converting the polygons to points for consistency. Using a 500-meter grid aligned with NTL resolution, we created separate grids for the commercial and industrial sectors. Employing spatial join, we merged commercial points with the commercial grids. Cells with at least one commercial point were labeled as commercial cells and later dissolved into a polygon file outlining the commercial area—this polygon file extracted commercial light sources from the NTL data. The exact process was performed to extract industrial NTL. As NTL radiance originates from mixed sources and we cannot discern pure sources, these results must be accepted as reasonable.

The monthly provincial cloud-free NTL (*cDN_p,m_*) datasets (Supplementary Figure S2) were aggregated to the national quarterly scale (*NTL_n,q_*) to align with the GDP data (Equation 5). Figure 1C presents the NTL used for the regression analyses. NTL tends to be highest in the second and fourth quarters because of the holiday seasons. Unlike EC, NTL tends to be lowest during the third quarter and the monsoon months.


(5) $$NTL_{n,q}=\overset{3}{\underset{m=1}{\sum }}\overset{n}{\underset{p=1}{\sum }}cDN_{p,m}\;\;$$

**Figure 2:**
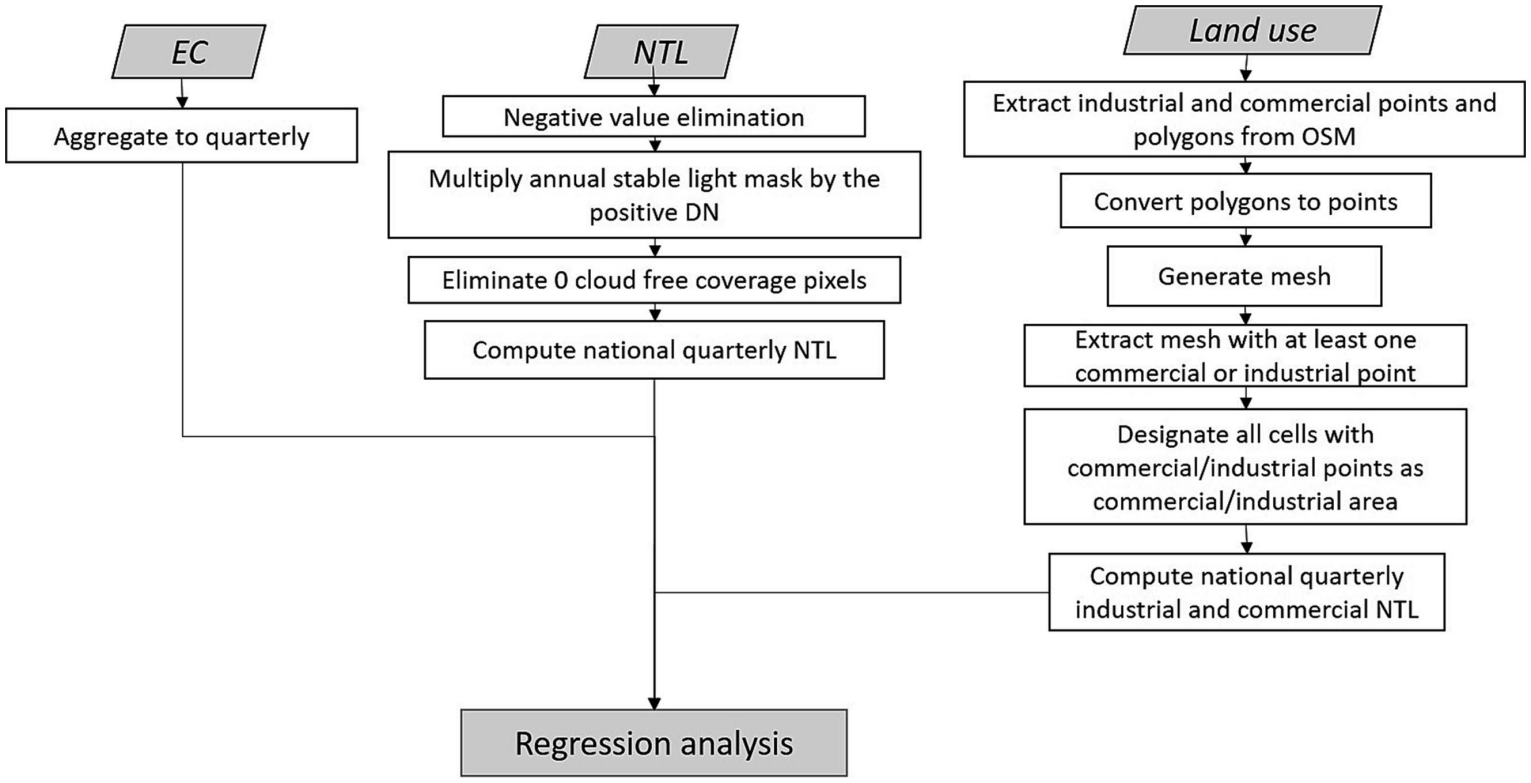
Data pre-processing for EC and NTL.

Preprocessing steps were executed using RStudio and ArcGIS Desktop 10.8.2. Figure 2 illustrates the preprocessing steps.

#### 2.1.4 Other data

Table 1 presents the independent variables used in the regression models. We collected the daily average temperature, maximum temperature, and humidity data from the Philippine Atmospheric, Geophysical, and Astronomical Services Administration (PAGASA). We computed the heat index using temperature and humidity data. Additionally, we aggregated daily weather data from daily into quarterly intervals (Supplementary Figures S3A,B). Data on the working age population, representing individuals aged 15–65, were obtained from PSA. Annual estimates for the overall and urban populations were collected from Macrotrends (Supplementary Figure S3C).

**Table 1:** Variables used in the regression models.

Variables (x)	Definitions
*ln GDP_t_*	Natural logarithm value of GDP at time t
*ln EC_t_*	Natural logarithm value of EC sum at time t
*ln NTL_t_*	Natural logarithm value of NTL sum at time t
*ln WorkDays_t_*	Natural logarithm value of number of working days per quarter
*ln WorkAgePop_t_*	Natural logarithm of working age population at time t
*ln Pop_t_*	Natural logarithm of total population at time t
*ln UrbPop_t_*	Natural logarithm of urban population at time t
*ln AveTemp_t_*	Natural logarithm of mean temperature at time t
*ln MaxTemp_t_*	Natural logarithm of maximum temperature at time t
*ln HI_t_*	Natural logarithm of the heat index at time t
*ln Humidity*	Natural logarithm of humidity at time t

The variables were aggregated into quarterly intervals to align with the GDP data. The datasets cover the period from quarter 2 of 2012 to quarter 4 of 2021.

### 2.2 Economic loss estimation

**Figure 3:**
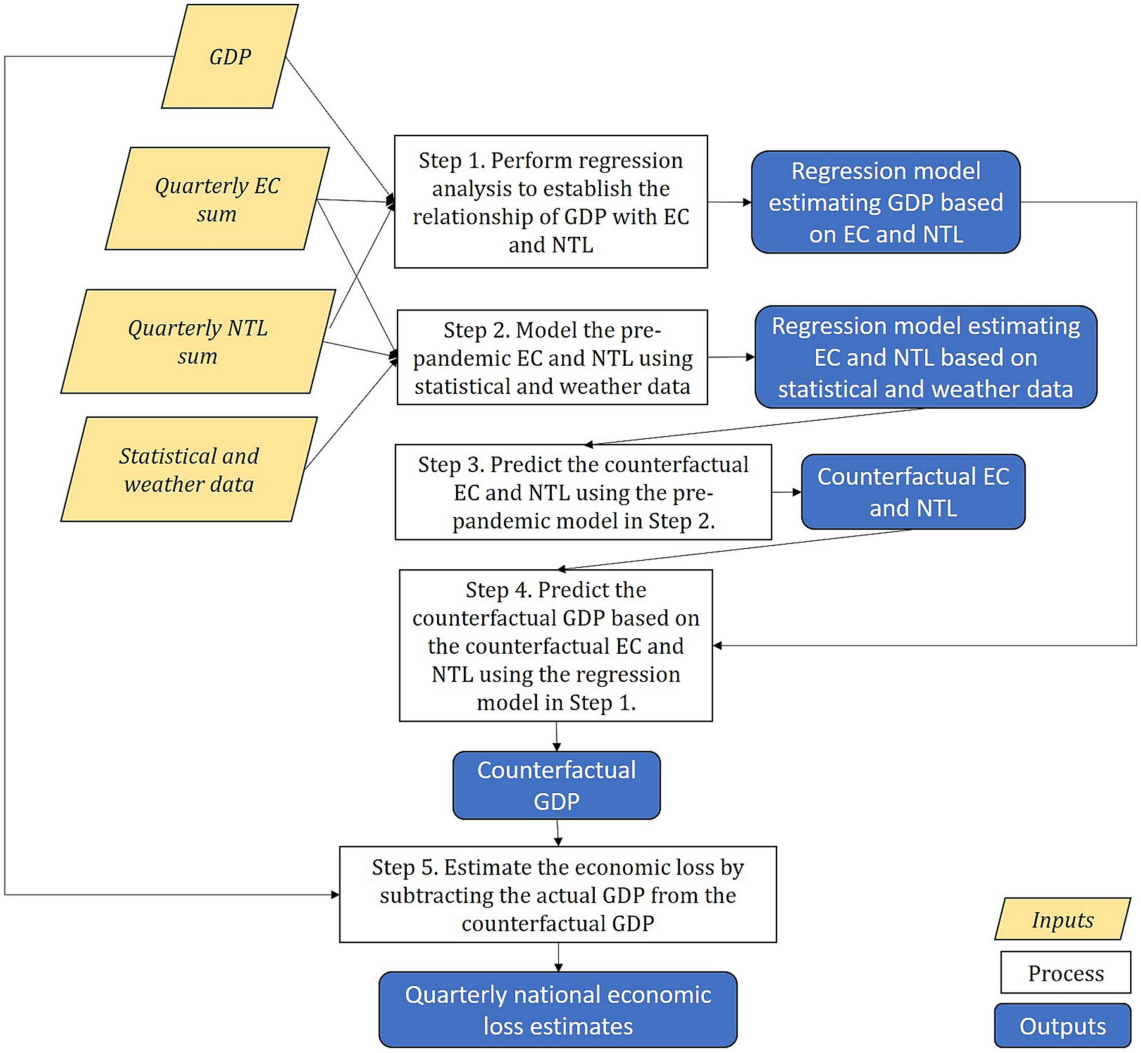
Research framework.

Figure 3 depicts the economic loss estimation process. Initially, we established the statistical relationship between GDP and the explanatory variables, namely EC and NTL, assessing their potential as substitutes for GDP if a strong relationship exists. Subsequently, we developed statistical models to forecast EC and NTL under a counterfactual scenario without COVID-19, incorporating weather conditions and statistical data. The next step involved projecting counterfactual GDP by utilizing the forecasted EC and NTL within the model. Finally, we estimated the economic losses attributed to COVID-19 by computing the difference between the actual and predicted GDPs. This identical method was employed to gauge the commercial and industrial economic losses due to the pandemic.

#### 2.2.1 Establishing the relationship of GDP with EC and NTL

Using the pre-pandemic data, we examined the relationship between GDP and EC and NTL to establish whether these variables can be strong substitutes for GDP, similar to a recent study (27). Equation 6 represents the regression of GDP on EC and NTL, aiming to determine the relationship among the variables. The model comprises GDP as the dependent variable regressed on EC and NTL, the trend variable $trend_{t}$ , and quarter dummy variable $q_{t}$ as independent variables. Similar to (27), variable $trend_{t}$ captures the time-related impact on GDP, while the quarter dummy variable $q_{t}$ captures seasonal variations across the GDP data.


(6) $$\ln GDP_{t}=\beta_{0}+\beta_{1}\ln EC_{t}+\beta_{2}\ln NTL_{t}+\gamma trend_{t}+\delta q_{t}+\varepsilon_{t}$$

where $\ln GDP_{t}$ , $\ln EC_{t}$ , and $\ln NTL_{t}$ are the natural logarithm of GDP, EC, and NTL at time t (measured in quarters), respectively. $\varepsilon_{t}$ is the error term, and $\beta_{0}$ , $\beta_{1}$ , $\beta_{2}$ , γ , and δ represent the parameters that must be estimated.

#### 2.2.2 Developing a statistical model for predicting counterfactual EC and NTL

Considering that EC and NTL have a reasonable relationship with GDP, we treated these explanatory variables as substitutes for GDP. With the predicted EC and NTL, we could estimate a counterfactual GDP. Hence, we formulated a statistical model to forecast EC and NTL, incorporating basic statistical factors and temperature. EC was regressed on the variables listed in Table 1, the trend variable $trend_{t}$ , and the quarter dummy variable $q_{t}$ . The selection of operational time and population is justified by their capacity to elucidate activity duration and intensity, respectively. Additionally, weather variables have been included due to their impact on EC, as explained by a previous study (28). Temperature variables dictate the extent of energy required for temperature regulation within a space. In accordance with a study (20) stating that urban density can influence NTL intensity, we also incorporated urban population figures and their ratio to signify economic activity. Including the trend and quarter dummy variables follows our approach to the GDP prediction models. The models for EC and NTL are expressed in Equations 7, 8, respectively.


(7) $$\ln EC_{t}=\beta_{0}+\beta x_{t}+\gamma trend_{t}+\delta q_{t}+\varepsilon_{t}$$

(8) $$\ln NTL_{t}=\beta_{0}+\beta x_{t}+\gamma trend_{t}+\delta q_{t}+\varepsilon_{t}$$

where $x_{t}$ is a vector of independent variables, and $\varepsilon_{t}$ is the error term. $\beta_{0}$ , γ , δ , and β denote the parameters that must be estimated. These models are estimated using data predating the onset of the COVID-19 pandemic.

#### 2.2.3 Predicting the counterfactual EC and NTL

To calculate the economic losses resulting from COVID-19, we subtracted the actual GDP from the projected counterfactual GDP following the onset of the pandemic (Equation 11). This computation involves forecasting counterfactual EC and NTL without COVID-19, utilizing the estimates for the independent variables specified in models 7 and 8. Specifically, we derived the counterfactual EC and NTL through Equations 9, 10.


(9) $$\ln cEC_{t}={\hat{\beta }}_{0}+\hat{\beta }x_{t}+\hat{\gamma }trend_{t}+\hat{\delta }q_{t}$$

(10) $$\ln cNTL_{t}={\hat{\beta }}_{0}+\hat{\beta }x_{t}+\hat{\gamma }trend_{t}+\hat{\delta }q_{t}$$

where $\ln cEC_{t}$ and $\ln cNTL_{t}$ represent the natural logarithm of the projected counterfactual EC and NTL, respectively, following the COVID-19 outbreak. The parameters ${\hat{\beta }}_{0}$ , $\hat{\beta }$ , $\hat{\gamma }$ , and $\hat{\delta }$ were estimated using data predating the COVID-19 outbreak.

#### 2.2.4 Predicting the counterfactual GDP

Subsequently, we employed the coefficients estimated in Equation 5 and plugged in the counterfactual EC and NTL values from Equations 9, 10 to estimate the counterfactual GDP, as determined by Equation 11.


(11) $$\ln cGDP_{t}={\hat{\beta }}_{0}+{\hat{\beta }}_{1}\ln cEC_{t}+{\hat{\beta }}_{2}\ln cNTL_{t}+\hat{\gamma }trend_{t}+\hat{\delta }q_{t}$$

where $\ln cGDP_{t}$ represents the natural logarithm of projected counterfactual GDP following the COVID-19 outbreak. Parameters ${\hat{\beta }}_{0}$ , ${\hat{\beta }}_{1}$ , ${\hat{\beta }}_{2}$ , $\hat{\gamma }$ , and $\hat{\delta }$ were estimated using the data preceding the COVID-19 outbreak.

#### 2.2.5 Estimating economic losses

**Figure 4:**
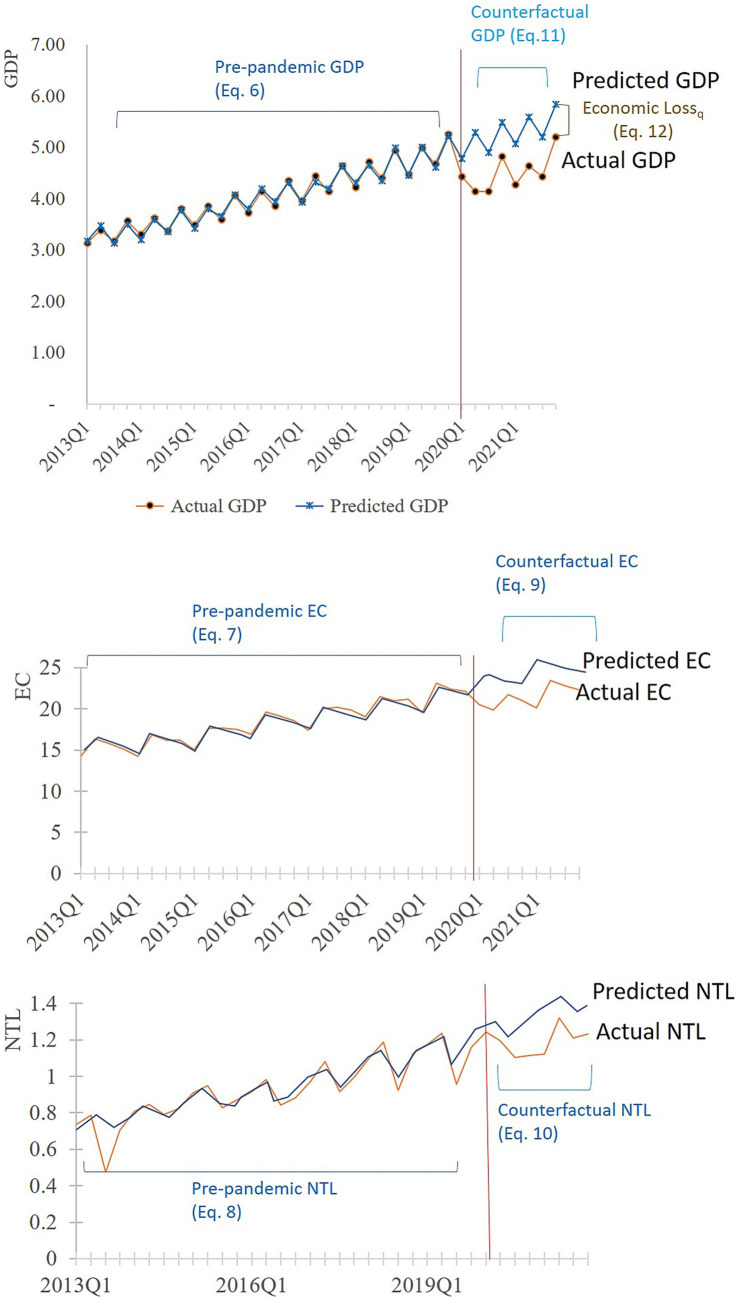
Illustration of the economic loss estimation process.

Finally, the economic loss resulting from COVID-19 was assessed using Equation 12.


(12) $$EconomicLoss_{t}=cGDP_{t}-aGDP_{t}$$

where $cGDP_{t}$ represents the exponential of $\ln cGDP_{t}$ , and $aGDP_{t}$ denotes the actual GDP obtained from official statistics. This process was replicated for estimating the commercial and industrial losses. The estimated commercial and industrial losses were adjusted to match the total economic losses. Figure 4 illustrates the economic loss estimation process.

## 3 Results

### 3.1 Relationship of GDP with EC and NTL

The results demonstrate that EC and NTL can be strong proxies for GDP (Table 2). Specifically, GDP has a stronger relationship with EC than with NTL. Models based on EC (GDPt1, GDPc1, GDPi1) have a higher coefficient of determination or *R*^2^ values than models based on NTL (GDPt2, GDPc2, GDPi2). In addition, EC’s coefficients were close to 1, which means that a unit increase in EC increases GDP from 1.1 to 1.3 units. In contrast, NTL’s coefficients ranged from 0.6 to 0.8. However, these models based on EC or NTL alone generally have higher Akaike information criterion (AIC). Models utilizing both EC and NTL (GDPt3, GDPc3, and GDPi3) had lower AIC and consistently exhibited higher *R*^2^ values than models solely relying on a single predictor. This suggests that combining EC with NTL slightly enhances the model’s strength. For the models combining EC and NTL, EC tended to have a greater effect on GDP. Note that in the case of model GDPc3, NTL displayed an insignificant *p*-value. For consistency, models GDPt3, GDPc3, and GDPi3 were employed to forecast the counterfactual GDP for 2020 and 2021, corresponding to the COVID-19 period.

**Table 2:** Regression model results for total, commercial, and industrial GDP using 2012 Q2 to 2019 Q4 data.

Coefficient (standard error)	GDPt1	GDPt2	GDPt3	GDPc1	GDPc2	GDPc3	GDPi1	GDPi2	GDPi3
Intercept	−2.545*** (0.448)	6.331*** (0.717)	−1.623** (0.541)	−4.752*** (0.549)	4.588** (1.305)	−4.473*** (0.569)	−5.632*** (0.593)	5.463** (1.241)	−5.340*** (0.528)
ln EC	1.064*** (0.027)		0.928*** (0.058)	1.255*** (0.036)		1.182*** (0.060)	1.260*** (0.038)		1.199*** (0.043)
ln NTL		0.640*** (0.052)	0.097* (0.037)		0.768*** (0.100)	0.065 (0.044)		0.704*** (0.103)	0.061** (0.0253)
Quarter 2	−0.061*** (0.011)	0.056 (0.031)	−0.046*** (0.011)	−0.033* (0.013)	0.113* (0.050)	−0.025 (0.014)	−0.029* (0.013)	0.053 (0.052)	−0.021 (0.012)
Quarter 3	−0.105*** (0.011)	0.144*** (0.033)	−0.070*** (0.017)	−0.058*** (0.013)	0.262*** (0.056)	−0.034 (0.021)	−0.177*** (0.014)	0.103 (0.059)	−0.139*** (0.017)
Quarter 4	0.026* (0.011)	0.146*** (0.031)	0.042** (0.011)	−0.008 (0.013)	0.129** (0.050)	0.001 (0.014)	0.030* (0.013)	0.128* (0.052)	0.041** (0.012)
*N*	31	31	31	31	31	31	31	31	31
Fes	Qtr	Qtr	Qtr	Qtr	Qtr	Qtr	Qtr	Qtr	Qtr
*R* ^2^	0.985	0.869	0.988	0.981	0.714	0.982	0.981	0.708	0.986
Adj- *R*^2^	0.983	0.849	0.986	0.978	0.670	0.979	0.978	0.663	0.983

Significance levels are * *p* < 0.05, ** *p* < 0.01, *** *p* < 0.001. The standard errors were reported inside the parentheses ().

The trend component was excluded from these models to mitigate multicollinearity issues with EC and NTL. The trend had a variance inflation factor (VIF) greater than 10. Removing the trend left NTL with the highest VIF: 6.8 in GDPt3, 3.3 in GDPi3, and 4.2 in GDPc3. The VIF for GDPt3 indicates a slight multicollinearity between EC and NTL. Nevertheless, the VIF was deemed acceptable as EC and NTL differ in seasonal patterns. Each of these contributed to explaining GDP differently.

### 3.2 Counterfactual models

Regression models were developed to estimate the counterfactual total, industrial, and commercial EC and NTL values (Table 3). Base models were developed for the Total EC (ECt1) and NTL (NTL1), Industrial EC (ECi1) and NTL (NTLi1), and Commercial EC (ECc1) and NTL(NTLc1). Meanwhile, the final models, denoted as ECt2, ECi2, ECc2, NTLt2, NTLi2, and NTLc2, incorporated only the significant variables. The ECt2, ECi2, and ECc2 models account for 99.3, 99.1, and 99.1% of the typical pre-COVID-19 total, industrial, and commercial EC, respectively. Conversely, the final NTL models explain 89.1, 81.1, and 81.6% of the typical pre-COVID-19 total, industrial, and commercial NTL, respectively. These models were employed to forecast the counterfactual EC and NTL values during COVID-19.

**Table 3:** Counterfactual EC and NTL regression models from 2012 quarter 2 to 2019 quarter 4.

Coefficient (standard error)	ECt1	ECt2	ECi1	ECi2	ECc1	ECc2	NTLt1	NTLt2	NTLi1	NTLi2	NTLc1	NTLc2
*Intercept*	79.146 (140.064)	−66.007*** (6.366)	325.644 (191.813)	319.739*** (73.589)	4.741 (158.779)	−58.314*** (6.609)	−755.150 (1257.702)	−282.2*** (44.57)	−1498.535 (1489.404)	−2639.291*** (620.029)	−1215.826 (1414.958)	−271.951*** (51.383)
*ln WorkDays*	−0.138 (0.111)		−0.147 (0.152)		−0.189 (0.126)		−0.142 (0.995)		−0.210 (1.178)		−0.265 (1.119)	
*ln WorkAgePop*	0.447 (0.329)	1.058*** (0.175)	0.188 (0.451)		0.711**·** (0.373)	1.085*** (0.182)	−2.304 (2.958)	−3.564** (1.239)	−3.094 (3.502)		−2.233 (3.327)	−4.494** (1.429)
*ln Pop*	14.858 (10.767)		32.869* (14.745)	25.957*** (4.630)	3.207 (12.205)		29.937 (96.679)		48.671 (114.490)		36.140 (108.767)	
*ln UrbPop*	−2.773 (4.918)		−4.477 (6.735)		0.989 (5.575)		−39.656 (44.163)		−81.262 (52.299)	−99.077*** (24.323)	−63.489 (49.684)	
*ln AveTemp*	−0.134 (1.655)		0.080 (2.267)		−0.351 (1.876)		−10.267 (14.862)		−4.659 (17.600)		−9.963 (16.721)	
*ln MaxTemp*	0.183 (0.108)	0.309** (0.106)	−0.010 (0.149)		0.280* (0.123)	0.359** (0.110)	0.317 (0.975)		0.275 (1.154)		0.497 (1.097)	
*ln Humidity*	−0.075 (0.286)		−0.266 (0.392)		0.013 (0.325)		−1.814 (2.572)		−1.729 (3.045)		−2.014 (2.893)	
*ln IH*	1.212 (1.410)		0.017 (1.931)		1.250 (1.599)		10.710 (12.664)	3.197 (1.720)	7.216 (14.997)		11.570 (14.247)	5.088* (1.983)
*trend*	−0.147 (0.149)	0.035*** (0.004)	−0.416 (0.205)	−0.389*** (0.079)	−0.039 (0.169)	0.030*** (0.004)	0.470 (1.341)	0.161*** (0.028)	1.033 (1.588)	2.185*** (0.521)	0.849 (1.509)	0.158*** (0.032)
Quarter 2	0.019 (0.028)	0.127*** (0.008)	0.074 (0.038)	0.0883*** (0.006)	0.013 (0.031)	0.111*** (0.008)	−0.189 (0.249)	−0.236 (0.180)	−0.319 (0.295)	0.041 (0.048)	−0.355 (0.280)	−0.466* (0.208)
Quarter 3	0.031 (0.023)	0.108*** (0.007)	0.101** (0.031)	0.099*** (0.006)	0.028 (0.026)	0.103*** (0.007)	−0.322 (0.202)	−0.375* (0.139)	−0.405 (0.240)	−0.209*** (0.048)	−0.446 (0.228)	−0.557** (0.160)
Quarter 4	0.056*** (0.014)	0.099*** (0.007)	0.084*** (0.019)	0.089*** (0.006)	0.058 (0.016)	0.104*** (0.008)	−0.021 (0.124)	−0.071 (0.082)	−0.038 (0.147)	0.021 (0.048)	−0.059 (0.140)	−0.132 (0.095)
N	31	31	31	31	31	31	31	31	31	31	31	31
Fixed effects	YES	YES	YES	YES	YES	YES	YES	YES	YES	YES	YES	YES
*R* ^2^	0.997	0.994	0.994	0.993	0.996	0.993	0.920	0.913	0.865	0.849	0.872	0.853
Adjusted *R*^2^	0.996	0.993	0.990	0.991	0.994	0.991	0.867	0.891	0.775	0.811	0.787	0.816

Significance levels are **·** *p* < 0.1, * *p* < 0.05, ** *p* < 0.01, *** *p* < 0.001. The standard errors were reported inside the parentheses ().

### 3.3 Economic losses

**Figure 5:**
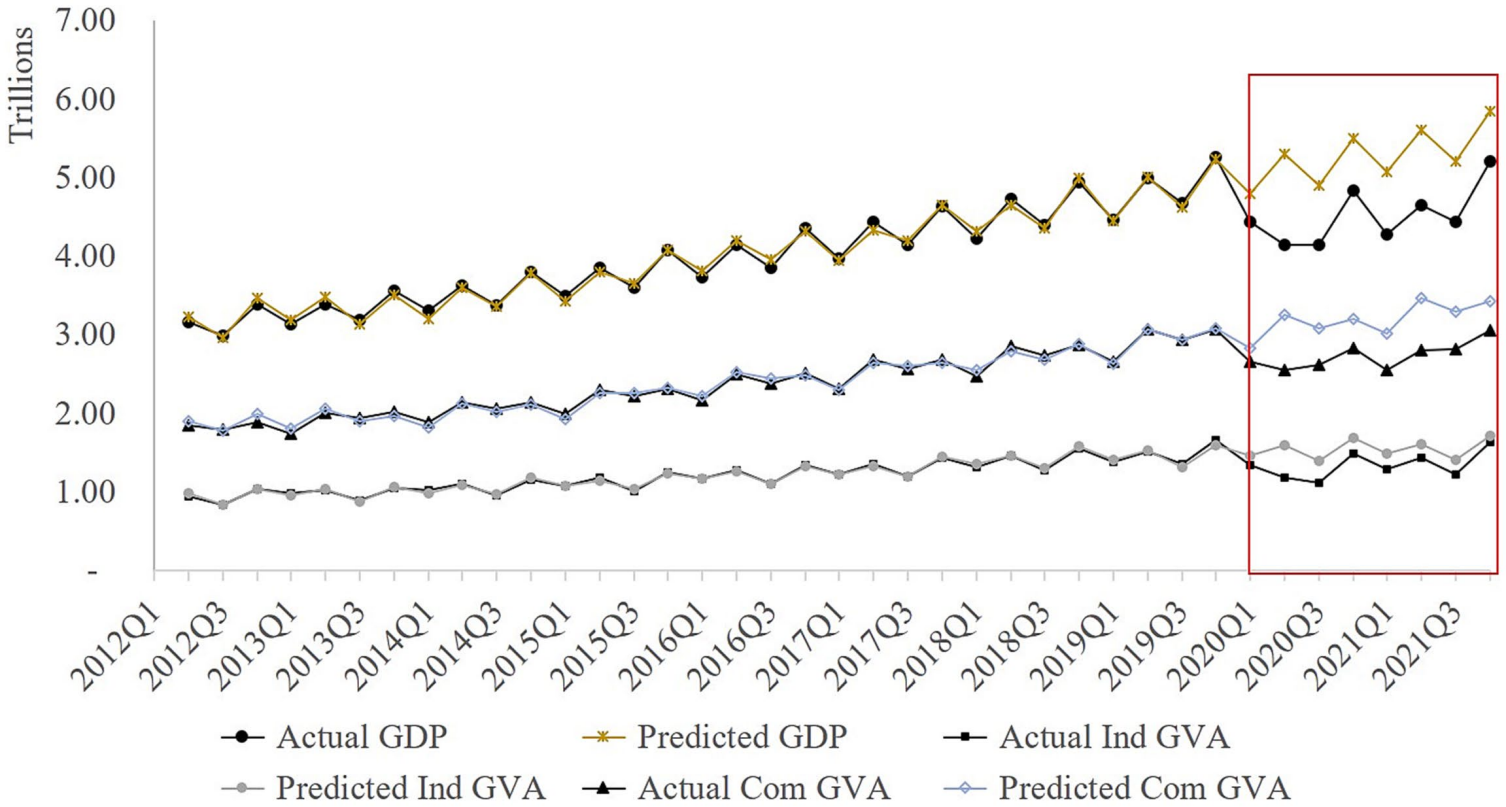
Actual and predicted GDP and GVA before and after the onset of COVID-19. The differences between the actual and predicted GDP and GVAs during the study period are highlighted in the red box.

The modeled GVAs and GDP closely followed the actual values leading up to 2019Q4, indicating a strong model fit (Figure 5). Furthermore, in the absence of COVID-19, the Total GDP, Industrial GVA, and Commercial GVA exhibited positive trends, peaking during the second and fourth quarters annually. However, as the COVID-19 lockdowns commenced in 2020Q1, actual values began to decline below their projected counterparts. The gaps between the counterfactual and actual values represent economic losses, with the most significant ones occurring in the second quarter of 2020, underscoring the profound impact of COVID-19 lockdowns on the economy. Total GDP and sectoral GVAs are yet to fully recover to the counterfactual values, signifying ongoing losses through the end of 2021.

**Figure 6:**
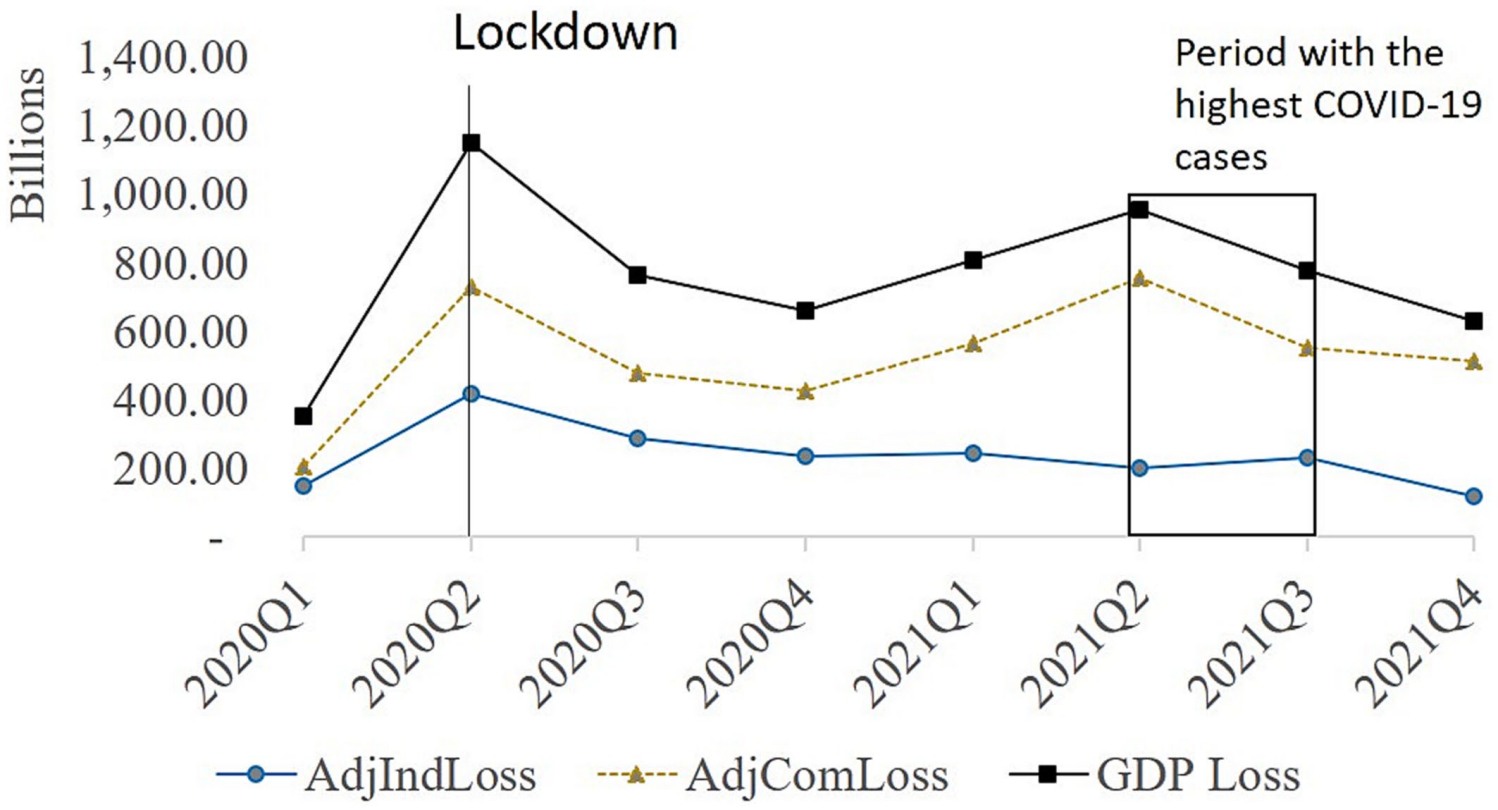
Quarterly economic activity losses for commercial and industrial sector in 2020 and 2021. Note the most stringent period is marked by the line and the peak of COVID-19 cases is high.

Across time, the highest total and sectoral losses occurred during 2020Q2, during the period of the strictest lockdown in the country (Table 4; Figure 6). Specifically, 2020Q2 witnessed the most Total GDP losses, amounting to approximately 1.15 trillion PhP (20.03 billion USD). Similarly, the industrial sector incurred substantial losses of 419.01 billion PhP (7.66 billion USD), while the commercial sector faced significant losses of 731.38 billion PhP (13.37 billion USD) during the same quarter. Notably, there were two peaks in both the national GDP loss and commercial sector losses, while the industrial sector demonstrated a single prominent peak.

**Table 4:** Estimated economic losses and loss rates per quarter from 2020 to 2021.

	GDP loss (billion PhP)	GDP loss rate	Adjusted industrial loss (billion PhP)	Industrial loss rate	Adjusted commercial loss (billion PhP)	Commercial loss rate
2020Q1^a^	350.79	0.07	147.82	0.10	202.96	0.07
2020Q2	1,150.46	0.22	419.08	0.26	731.38	0.22
2020Q3	764.92	0.16	285.48	0.21	479.44	0.16
2020Q4^b^	661.67	0.12	234.27	0.14	427.40	0.13
2021Q1	807.18	0.16	242.51	0.16	564.67	0.19
2021Q2	955.98	0.17	199.53	0.12	756.46	0.22
2021Q3	779.19	0.15	229.03	0.16	550.16	0.17
2021Q4	631.34	0.11	116.16	0.07	515.19	0.15
2020	2,927.84	0.14	1,086.65	0.16	1,841.19	0.14
2021	3,173.69	0.15	787.23	0.11	2,386.46	0.15

a Includes damage and loss from Taal Volcano Eruption amounting to 8.4 billion PhP (41).

b Covers the damage and loss from Typhoon Goni and Vamco, estimated to be 105 billion PhP (41).

Across sectors, the commercial sector experienced more substantial losses, totaling 1.84 trillion PhP (33.66 billion USD) inw2020 and 2.39 trillion PhP (43.63 billion USD) in 2021, compared to the industrial sector’s losses of 1.09 and 0.79 billion PhP (19.87 and 14.39 billion USD) during the same respective years. Consequently, 69% of the total losses were attributed to the commercial sector, while the industrial sector accounted for the remaining 31%. Notably, despite its overall losses, the industrial sector suffered a more significant loss rate, representing 26% compared to the commercial sector’s 19%, especially during 2020Q2. Nevertheless, there are signs of faster recovery within the industrial sector.

## 4 Discussion

### 4.1 Economic losses in the Philippines

In the Philippines, economic losses due to COVID-19 in 2020 and 2021 amounted to 2.9 and 3.2 trillion PhP, respectively. This study’s 2020 estimate falls within the range of economic losses reported in previous studies (12, 13, 29). A study reported 2.1 trillion PhP losses covering 2 years under the assumption of uninterrupted recovery from October 31, 2020 (13). Our results update this previous study’s estimate by including the effect of succeeding lockdowns resulting in higher losses in 2021 attributed to further economic disruptions from lockdowns and viral transmission. The National Economic and Development Authority estimated 4.3 trillion PhP in COVID-19 losses in 2020. This estimate is higher than our results, but the study acknowledged the possible long-term impacts of around 40 trillion in the next four decades (29). Reports state that 2020 suffered the worst economic loss on record (30). This study’s economic cost estimates show that COVID-19 caused losses incomparable with any other destructive events in the country’s record. Compared with other countries (7), our study’s 2020 loss estimate was higher than that of China’s, which amounted to 42.2 billion USD, but lower than that of Germany’s losses, which amounted to 56.4 billion USD.

### 4.2 Economic losses across time and sectors

As expected, economic losses peaked during the lockdown. The national GDP’s initial and more pronounced peak occurred during the second quarter of 2020 (Figure 6), coinciding with the stringent ECQ measures (31) (Supplementary Figure S4). The second peak materialized during the second quarter of 2021, aligning with an upsurge in COVID-19 (32) cases despite the quarantine stringency remaining constant.

Distinct loss patterns emerged between the two sectors, particularly in the triggers for commercial and industrial losses. The industrial sector loss lacked a discernible second peak, indicating that it was less severely affected by the rise in COVID-19 cases and mostly affected by the lockdown implementation. In contrast, the commercial sector’s loss paralleled that of the total GDP, indicating that both lockdowns and viral transmission affected it.

It follows that commercial losses surpassed industrial losses. Business interruptions affected more establishments and employees in the commercial sector, representing over 60% of the GDP, over 85% of operating establishments, and 75% of employment. Mobility data (33) also supports this finding through the evidence of lowered human presence in the commercial sector, recreational places, transit stations, and workplaces (Supplementary Figure S5). Furthermore, unlike industrial operations, consumer behavior influenced commercial productivity (34); for example, buyers prioritized essential expenditures and preferred online purchases. Moreover, households facing unemployment and income reduction tended to reduce spending on non-essential items (3). Finally, social distancing measures controlled the number of customers in establishments, effectively reducing commercial activities.

This study’s contribution revolves around analyzing actual losses and their temporal and sectoral distribution. Understanding this temporal dimension is crucial for formulating policies that balance health and economy during pandemics. However, economic impacts are often reported without the temporal dimension due to data limitations. The use of EC and NTL data facilitated this temporal analysis. Moreover, these datasets enabled sectoral loss comparison, serving as a basis for financial support allocation.

### 4.3 Limitations and recommendations

This study focused on the commercial and industrial sectors and excluded agricultural losses as agricultural activities were permitted during the lockdown, resulting in agricultural growth. Further, this method is unsuitable for estimating agricultural losses because EC does not specify energy used for agricultural activities, and agricultural areas have lower light emissions. Additionally, this study did not include residential losses considering residential EC increased during the lockdown, as evidenced by increased mobility in residential areas (Supplementary Figure S5).

Another limitation is that total, industrial, and commercial losses were modeled separately. The commercial and industrial losses had to be adjusted based on their ratios to the total to ensure the sum would add up to the total loss. To address this limitation, future research can refine models by incorporating the sectors in one model.

Note that the economic impacts from other hazards within the study period, including the Taal Volcano eruption on January 12, 2020, amounting to 8.4 billion PhP (35) loss, and Typhoons Goni and Vamco in November 2020, costing 105 billion PhP (35), were included in the estimated economic losses.

Further, NTL image resolution and lack of comprehensive land use data limited the accuracy of NTL attribution. If used on a provincial or municipal scale, existing land use maps could be used to improve the industrial and commercial NTL accuracy. Future research may also use NTL and EC data to examine the spatial distribution of economic losses.

This study demonstrated that EC and NTL are strong proxies for GDP. Future studies may use finer-resolution data to examine the losses at a greater spatial and temporal scale. Finally, pandemic-related policies should consider the temporal dimension of economic losses to better balance economic and health concerns during such situations.

## Data availability statement

The original contributions presented in the study are included in the article/Supplementary material, further inquiries can be directed to the corresponding author.

## Author contributions

MD: Conceptualization, Data curation, Formal analysis, Investigation, Methodology, Validation, Visualization, Writing – original draft, Writing – review & editing. TF: Conceptualization, Methodology, Supervision, Writing – original draft, Writing – review & editing, Resources. HT: Conceptualization, Resources, Writing – review & editing.

## Footnotes

^1^ https://eogdata.mines.edu/

## References

[1] Chen S Igan DO Pierri N Presbitero AF. Tracking the economic impact of COVID-19 and mitigation policies in Europe and the United States: IMF Working Paper. (2020). Available at: https://www.imf.org/en/Publications/WP/Issues/2020/07/10/Tracking-the-Economic-Impact-of-COVID-19-and-Mitigation-Policies-in-Europe-and-the-United-49553 (Accessed June 30, 2023).

[2] Naseer S Khalid S Parveen S Abbass K Song H Achim MV. COVID-19 outbreak: impact on global economy. Front Public Health. (2023) 10:1–13. doi: 10.3389/fpubh.2022.1009393, PMID: 36793360 PMC9923118

[3] Ajmal MM Khan M. The global economic cost of coronavirus pandemic: current and future implications. Public Admin Policy. (2021) 24:290–305. doi: 10.1108/PAP-10-2021-0054

[4] Nundy S Ghosh A Mesloub A Albaqawy GA Alnaim MM. Impact of COVID-19 pandemic on socio-economic, energy-environment and transport sector globally and sustainable development goal (SDG). J Clean Prod. (2021) 312:127705–25. doi: 10.1016/j.jclepro.2021.127705, PMID: 36471816 PMC9710714

[5] Congressional Research Service. Global economic effects of COVID-19. (2021). Available at: https://sgp.fas.org/crs/row/R46270.pdf (Accessed October 12, 2022).

[6] Gopinath G. The great lockdown: worst economic downturn since the Great Depression. (2020). Available at: https://www.imf.org/en/Blogs/Articles/2020/04/14/blog-weo-the-great-lockdown-worst-economic-downturn-since-the-great-depression (Accessed July 21, 2023).

[7] Zhang HL You S Zhang M Chen A Hu Z Liu Y. Empirical study of monthly economic losses assessments for “standard unit lockdown” due to COVID-19. Front Public Health. (2022) 10:1–11. doi: 10.3389/fpubh.2022.859751, PMID: 35619804 PMC9129268

[8] Hapal K. The Philippines’ COVID-19 response: securitising the pandemic and disciplining the Pasaway. J Curr Southeast Asia Aff. (2021) 40:224–44. doi: 10.1177/1868103421994261

[9] Philippine Statistics Authority. Quarterly National Accounts Linked Series (Q1 2000 to Q4 2023). (2024). Available at: https://psa.gov.ph/statistics/national-accounts/data-series (Accessed February 8, 2024).

[10] Panopio EL Cudia CP. Impact of COVID-19 on the 2020 financial results of selected Philippine publicly-listed companies. DLSU Bus Econ Rev. (2022) 32:92–102.

[11] Shinozaki S Rao L. COVID-19 impact on micro, small, and medium-sized enterprises under the lockdown: Evidence from a rapid survey in the Philippines. (2021). Available at: https://www.adb.org/sites/default/files/publication/677321/adbi-wp1216.pdf (Accessed October 12, 2022).

[12] Santos JR Tapia JFD Lamberte A Solis CA Tan RR Aviso KB. Uncertainty analysis of business interruption losses in the Philippines due to the COVID-19 pandemic. Economies. (2022) 10:202. doi: 10.3390/economies10080202

[13] Yu KDS Aviso KB Santos JR Tan RR. The economic impact of lockdowns: a persistent inoperability input-output approach. Economies. (2020) 8:109. doi: 10.3390/economies8040109

[14] de Lara-Tuprio EP Eastuar MRJE Sescon JT Lubangco CK Castillo RCJT Teng TRY. Economic losses from COVID-19 cases in the Philippines: a dynamic model of health and economic policy trade-offs. Humanit Soc Sci Commun. (2022) 9:111. doi: 10.1057/s41599-022-01125-4

[15] Yu ES Choi JY. The causal relationship between energy and GNP: an international comparison. J Energy Dev. (1985) 10:249–72.

[16] Fatai K Oxley L Scrimgeour FG. Modelling the causal relationship between energy consumption and GDP in New Zealand, Australia, India, Indonesia, the Philippines and Thailand. Math Comput Simul. (2004) 64:431–45. doi: 10.1016/S0378-4754(03)00109-5

[17] Doll CNH Muller JP Elvidge CD. Night-time imagery as a tool for global mapping of socioeconomic parameters and greenhouse gas emissions. Ambio. (2000) 29:157–62. doi: 10.1579/0044-7447-29.3.157

[18] Ghosh T Anderson SJ Elvidge CD Sutton PC. Using nighttime satellite imagery as a proxy measure of human well-being. Sustainability. (2013) 5:4988–5019. doi: 10.3390/su5124988

[19] Henderson JV Storeygard A Weil DN. Measuring economic growth from outer space. Am Econ Rev. (2012) 102:994–1028. doi: 10.1257/aer.102.2.994, PMID: 25067841 PMC4108272

[20] Wu J Wang Z Li W Peng J. Exploring factors affecting the relationship between light consumption and GDP based on DMSP/OLS nighttime satellite imagery. Remote Sens Environ. (2013) 134:111–9. doi: 10.1015/j.rse.2013.03.001

[21] Pagaduan JA. Do higher-quality nighttime lights and net primary productivity predict subnational GDP in developing countries? Evidence from the Philippines. Asian Econ J. (2022) 36:288–317. doi: 10.1111/asej.12278

[22] Strobl E. The impact of typhoons on economic activity in the Philippines: Evidence from Nightlight Intensity. ADB Economics Working Paper Series. (2019). Available at: https://www.adb.org/publications/impact-typhoons-philippines (Accessed July 11, 2023).

[23] Cheng Y Han X. Assessing the economic loss due to natural disasters from outer space. Clim Serv. (2022) 26:100286–8. doi: 10.1016/j.cliser.2022.100286

[24] Elvidge CD Baugh K Zhizhin M Hsu FC Ghosh T. VIIRS night-time lights. Int J Remote Sens. (2017) 38:5860–79. doi: 10.1080/01431161.2017.1342050

[25] Liu H Luo N Hu C. Detection of county economic development using LJ1-01 nighttime light imagery: a comparison with NPP-VIIRS data. Sensors. (2020) 20:22. doi: 10.3390/s20226633, PMID: 33228106 PMC7699407

[26] Herfort B Lautenbach S Porto de Albuquerque J Anderson J Zipf A. A spatio-temporal analysis investigating completeness and inequalities of global urban building data in OpenStreetMap. Nat Commun. (2023) 14:3985. doi: 10.1038/s41467-023-39698-6, PMID: 37414776 PMC10326063

[27] Beyer RCM Franco-Bedoya S Galdo V. Examining the economic impact of COVID-19 in India through daily electricity consumption and nighttime light intensity. World Dev. (2021) 140:105287. doi: 10.1016/j.worlddev.2020.105287, PMID: 34305264 PMC8294606

[28] Baduyan-Dacuycuy C. Energy consumption, weather variability and gender in the Philippines: a discrete/continuous approach. (2017). Available at: https://pidswebs.pids.gov.ph/CDN/PUBLICATIONS/pidsdps1706.pdf (Accessed December 27, 2022).

[29] National Economic and Development Authority. COVID-19 pandemic to cost PhP 41.4 T for the next 40 years – NEDA. (2021). Available at: https://neda.gov.ph/covid-19-pandemic-to-cost-php-41-4-t-for-the-next-40-years-neda/ (Accessed October 2, 2023).

[30] Venzon C. Philippines GDP shrinks 9.5% in 2020, worst since 1947. Nikkei Asia. (2021). Available at: https://asia.nikkei.com/Economy/Philippines-GDP-shrinks-9.5-in-2020-worst-since-1947(Accessed February 8, 2024).

[31] Hale T Angrist N Goldszmidt R Kira B Petherick A Phillips T. A global panel database of pandemic policies Oxford COVID-19 government response tracker. Nat Hum Behav. (2021) 5:529–38. doi: 10.1038/s41562-021-01079-8, PMID: 33686204

[32] Department of Health. DOH COVID Data Drop. (2022). Available at: https://data.gov.ph/index/public/dataset/ (Accessed December 4, 2022)

[33] Google LLC. Google COVID-19 community mobility reports. (2021). Available at: https://www.google.com/covid19/mobility/ (Accessed September 12, 2022).

[34] Tao H Sun X Liu X Tian J Zhang D. The impact of consumer purchase behavior changes on the business model design of consumer services companies over the course of COVID-19. Front Psychol. (2022) 13:818845. doi: 10.3389/fpsyg.2022.818845, PMID: 35310236 PMC8927628

[35] Laforga BM. Damage from November typhoons, eruption of Taal in 2020 reckoned at P113 billion. Business world. (2021). Available at: https://www.bworldonline.come/editors-picks/2021/03/04/348398/damage-from-november-typhoons-eruptions-of-taal-in-2020-reckoned-at-p113-billion/ (Accessed October 2, 2023).

